# P-700. Surveillance of F-Gene Sequences from hMPV Isolates Reveals a Dominance of Subtype A2 and Absence of A1 in Contemporary Isolates

**DOI:** 10.1093/ofid/ofaf695.912

**Published:** 2026-01-11

**Authors:** Vancheswaran Gopalakrishnan, Emma Schaefer, Tyler M Brady, Joe Francica, Anna Kushnir, Ondrej Podlaha, Taylor Cohen, Anastasia A Aksyuk, Kevin M Tuffy

**Affiliations:** AstraZeneca, Gaithersburg, MD; AstraZeneca, Gaithersburg, MD; AstraZeneca, Gaithersburg, MD; AstraZeneca, Gaithersburg, MD; AstraZeneca, Gaithersburg, MD; AstraZeneca, Gaithersburg, MD; AstraZeneca, Gaithersburg, MD; Translational Medicine, Vaccines & Immune Therapies, BioPharmaceuticals R&D, AstraZeneca, Gaithersburg, MD, USA, Gaithersburg, MD; AstraZeneca, Gaithersburg, MD

## Abstract

**Background:**

Human metapneumovirus (hMPV) is a common cause of respiratory tract infections and is associated with severe disease among high-risk populations: neonates, young children, older adults, and immunocompromised individuals. Since surveillance data for hMPV are limited, immediate attention is warranted to guide therapeutic programs for hMPV.

**Figure:**
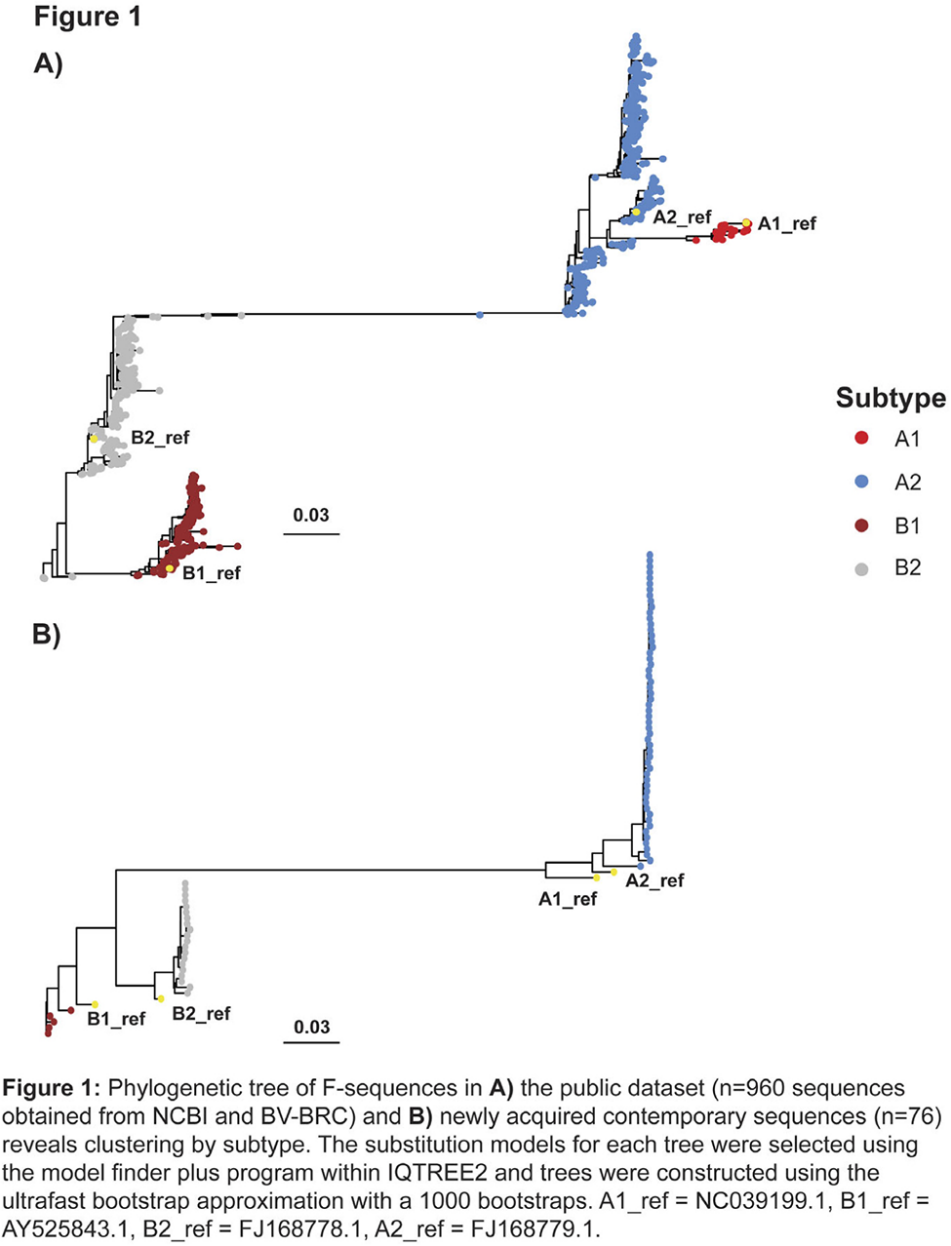

**Methods:**

960 hMPV F-gene sequences collected between 1982 and 2023 were sourced from NCBI and BV-BRC and subtyped based on minimum phylogenetic distance to determined reference strains. Additionally, 76 contemporary hMPV F sequences from hMPV-positive remnant nasal specimens collected at two US sites between 2022 and 2023 were obtained. F-gene sequences from nasal specimens were amplified by PCR and sequenced using the 2x150bp protocol on the Illumina platform. Subtypes were assigned based on the highest alignment score for each subtype’s consensus against its respective reference sequence. Variant calling for both datasets was preformed using RADAR, an internally developed tool.

**Results:**

The public sequence dataset (N=960) included 264 sequences collected from 2017–present (contemporary) and the top 4 depositing countries were Australia, Netherlands, Japan, and US. The A2 subtype was the most prevalent overall (47.9%) and among contemporary sequences (51.1%) (Figure 1A). In contrast, the A1 subtype had the least prevalence overall (3.3%) and was not detected in the contemporary dataset. The B1 (25.4%) and B2 (23.5%) subtypes were equally prevalent in the contemporary dataset. Among the 76 newly acquired contemporary US hMPV F sequences, 32 were obtained from patients older than 18 years, with an equal representation of males and females. The distribution of subtypes in these sequences mirrored that of the public contemporary dataset, with 67% assigned to A2 and 0% to A1 (Figure 1B). Additionally, we evaluated the degree of F-protein conservation across hMPV subtypes and noted an average conservation greater than 98% for all 4 subtypes, with the most prevalent variants also being consistently observed in both datasets.

**Conclusion:**

Our work establishes a robust baseline for hMPV surveillance. It underscores the importance of the A2 subtype in guiding therapeutic efforts and highlights the F-protein as an attractive therapeutic candidate.

**Disclosures:**

Vancheswaran Gopalakrishnan, PhD, AstraZeneca: Employment|AstraZeneca: Stocks/Bonds (Public Company) Emma Schaefer, BSc, AstraZeneca: Employment|AstraZeneca: Stocks/Bonds (Public Company) Tyler M. Brady, MPH, AstraZeneca: Employment|AstraZeneca: Stocks/Bonds (Public Company) Joe Francica, PhD, AstraZeneca: Employment|AstraZeneca: Stocks/Bonds (Public Company) Anna Kushnir, PhD, AstraZeneca: Employment Ondrej Podlaha, PhD, AstraZeneca: Employment|AstraZeneca: Stocks/Bonds (Public Company) Taylor Cohen, PhD, AstraZeneca: Employment|AstraZeneca: Stocks/Bonds (Public Company) Anastasia A. Aksyuk, PhD, AstraZeneca: Employment|AstraZeneca: Stocks/Bonds (Public Company)|MesoScale Diagnostics: Intellectual Property/Patents Kevin M. Tuffy, MS, AstraZeneca: Employment|AstraZeneca: Stocks/Bonds (Public Company)

